# Metagenomic 18S rDNA reads revealed zonation of eukaryotic communities in the Yongle blue hole

**DOI:** 10.3389/fmicb.2024.1420899

**Published:** 2024-07-29

**Authors:** Hongxi Zhang, Taoshu Wei, Qingmei Li, Liang Fu, Manjie Li, Lisheng He, Yong Wang

**Affiliations:** ^1^Institute of Deep Sea Science and Engineering, Chinese Academy of Sciences, Sanya, China; ^2^University of Chinese Academy of Sciences, Beijing, China; ^3^Sansha Track Ocean Coral Reef Conservation Research Institute Co., Ltd., Sansha, China; ^4^Institute for Ocean Engineering, Shenzhen International Graduate School, Shenzhen, China; ^5^Shenzhen Key Laboratory of Advanced Technology for Marine Ecology, Shenzhen International Graduate School, Tsinghua University, Shenzhen, China

**Keywords:** Yongle blue hole, 18S rRNA gene, eukaryotic communities, anoxic water, metagenomics

## Abstract

The Yongle blue hole (YBH), situated in the South China Sea, represents a compelling subject of study in marine microbiology due to its unique redox-layered microbial ecosystems. However, the diversity and ecology of microbial eukaryotes within the YBH remains underexplored. This study endeavors to bridge this gap through the application of the *in situ* microbial filtration and fixation (ISMIFF) device to collect 0.22–30 μm microbial samples from 21 water layers of YBH. Subsequent extraction of 18S rRNA metagenomic reads of 21 metagenomes and 10 metatranscriptomes facilitated a comprehensive analysis of community structures. Findings revealed a pronounced superiority in the diversity and richness of eukaryotic microorganisms in the oxic zone compared to its suboxic and anoxic counterparts. Notably, Dinophyceae and Maxillopoda emerged as the predominant taxa based on the analysis of the 18S rRNA reads for the V4 and V9 regions, which showed stratification In their relative abundance and suggested their potential role in the thermo-halocline boundaries and oxic-anoxic interface. Specifically, In these eukaryotic microbial communities, Dinophyceae exhibited significant abundance at 20 m (20.01%) and 105 m (26.13%) water depths, while Maxillopoda was prevalent at 40 m (22.84%), 80 m (23.19%), and 100 m (15.42%) depths. A part of these organisms, identified as larvae and protists, were likely attracted by swarming chemosynthetic bacterial prey prevailing at the thermo-halocline boundaries and oxic-anoxic interface. Furthermore, the phylogenetic relationships of the major 18S operational taxonomic units (OTUs) showed a close adjacency to known species, except for three Dinophyceae OTUs. In conclusion, this study provides critical insights into the vertical distribution and transcriptional activity of <30-μm eukaryotic microbes, shedding light on the taxonomic novelty of eukaryotic microorganisms within the semi-enclosed blue holes.

## 1 Introduction

The Yongle blue hole (YBH), situated in the Xisha Islands of the South China Sea, presents a stratified aqueous environment comprising a superficial oxic layer, a relatively stable oxic-anoxic interface, and a deep anoxic layer (Yao et al., 2018; Xie et al., 2019), with a water depth of 300.89 m (Yao et al., 2020). Water below approximately 100 m, exhibiting seasonal fluctuations in depth, and contains a significant amount of hydrogen sulfide and methane, likely resulting from biodegradation (Xie et al., 2019). The unique topographical structure of the cave acts as a barrier to vertical mixing, preventing the transport of external seawater and particulate organic matter to the cave’s bottom (Li et al., 2018, 2020; Gao et al., 2022). Temperature and salinity within the water column exhibit distinct stratification, and remain relatively constant in the deeper layer (Xie et al., 2019). This results in a unique physical–chemical characteristics, such as a strong thermo-halocline, a highly stratified water column, and thick anoxic and hydrogen sulfided layers. Such conditions are believed to support the development of a distinct ecosystem within the YBH. The stable anoxic environment provided by the YBH serves as an important model for studying ancient anoxic oceans and the early evolutionary history of eukaryotes (Li et al., 2010; Mentel and Martin, 2010; Zhang et al., 2024).

Eukaryotic organisms residing beneath the redox layer encounter environmental conditions markedly distinct from those in the oxygen-rich water layer (Behnke et al., 2006). The formation of symbiotic associations among the domains Bacteria, Archaea, and Eukarya in anoxic water columns is reported to be prevalent, positing a potential adaptation mechanism for eukaryotic microbes to exploit ecologically challenging habitats (Orsi et al., 2012). Surveys of marine blue holes have revealed numerous new species, including various types of crustaceans and foraminifera (Lejeusne and Chevaldonné, 2010; Boxshall and Jaume, 2012). Crustaceans have been found to dominate in some blue holes in the Bahamas and the coast of Mexico with a significant biodiversity, with many species unique to blue holes and not found in the surrounding waters (Kornicker et al., 2007; Iliffe and Kornicker, 2009). The interior of the holes contains a large number of planktons that are significantly different from those found on the surrounding outer reef slope, exhibiting notably higher biodiversity and abundance (Chen et al., 2018). Due to the challenges of studying the morphological and ultrastructural characteristics of microbial eukaryotes, analytical methods based on 18S rRNA gene clone libraries have been used for taxonomic analysis in different marine ecosystems (Zuendorf et al., 2006; Behnke et al., 2010; Wang et al., 2014). Using amplicons of 18S rRNA genes, the microbial community structures in the water column at different depths of YBH were preliminarily analyzed (Liu et al., 2019). It was observed that the abundance of eukaryotic microbes in the cave is significantly higher than that in the outer reef slope, alongside a large number of endemic species have been found, indicating the need for further study (Liu et al., 2019). It is challenging to recover intact eukaryotic microbial cells from the deep anoxic water layer (Wylezich and Jürgens, 2011) and, furthermore, there have been observations of biases introduced in the analysis of the community structure when using18S rRNA gene amplicons (Yeh et al., 2021). To address these challenges, metagenomics, based on the extraction of 18S ribosomal DNA reads from metagenomes, has been used to study microbial diversity and overcome these issues (Logares et al., 2014). This method allows the detection of low-abundance taxa and has been applied in the analysis of bathypelagic microbial eukaryotes (Pernice et al., 2016). It has been noted that the microbial eukaryote communities of the anoxic and suboxic water column are primarily influenced by oxygen concentration, as well as the sampling method (Duret et al., 2015). The sampling method employed could have affected the microorganisms in the deep layers of YBH with low oxygen and light density (Torres-Beltran et al., 2019). An *in situ* microbial filtration and fixation (ISMIFF) apparatus, which is mounted on manned submersibles, remotely operated vehicles (ROVs), and landers, has the capability to operate automatically in the full ocean depth (Gao et al., 2019; Wang et al., 2019; Wei et al., 2020). This technology was utilized to enrich and fix microorganisms in YBH *in situ*, allowing for a better understanding of the community structure and *in situ* metabolism of prokaryotic microbes without being impacting by environmental changes during sampling (Zhang et al., 2023, 2024).

The pico- and nano-sized (0.22–20 μm) eukaryotes (Ceccarelli et al., 2013) play a crucial role in marine plankto ecosystems as both primary producers and major bacterial grazers (Li et al., 2023). Also, the 0.2–30 μm diameter fraction includes pico- (0.2–2 μm), nano- (2–20 μm), and small microplankton (20–30 μm), representing a wide range of protistan species that are studied to analyze the structure of planktonic microbial eukaryotic communities (David et al., 2021). Nanoeukaryotes form part of the diet for some plankton larvae, impacting the community structure of eukaryotic microorganisms (Lindeque et al., 2015). In this study, we employed 18S rRNA metagenomic Illumina reads (miTags) to investigate the eukaryotic communities in the water column of YBH, which provides evidence for the uniqueness and distribution of eukaryotic species in suboxic and anoxic zones of blue holes.

## 2 Materials and methods

### 2.1 Sampling

All samples were obtained from YBH (16°31′30″N; 111°46′05″E) in the South China Sea. Water samples were collected by the ISMIFF apparatus (Wang et al., 2019) at 21 depths from the surface to the bottom layer inside the YBH. In the April cruise, 16 samples were collected in the depth range of 1–290 m (Supplementary Table S1). In the September cruise, we collected samples from five additional suboxic and anoxic water layers that were not sampled in April, including depths of 96.5 m, 98.5 m, 99.5 m, 100.5 m, and 250 m. ISMIFF was used to filter ~30–60 L seawater by a 0.22-μm pore size polycarbonate membrane (Millipore, Massachusetts, United States) with a prefiltration through a 30-μm mesh net. Immediately after ISMIFF was recovered, the membrane was taken out from the filtration chamber and put into a 15 mL centrifuge tube containing 3 mL RNAlater (Ambion, Carlsbad, CA, United States). The tube was then frozen in liquid nitrogen immediately and stored at −80°C until further processing. All operations were completed within 30 min. An SBE 37SMP-ODO conductivity-temperature-depth (CTD) unit and a dissolved oxygen (DO) sensor (Sea-Bird, Bellevue, WA, United States) were used to detect the temperature, salinity, and DO. pH was measured directly with Hach model HQ 40d portable combination meter (HACH, Loveland, CO, United States).

### 2.2 Nucleic acids extraction, metagenome and metatranscriptome sequencing

Total genomic DNA and RNA were extracted from 21 ISMIFF samples using the DNA/RNA co-extraction kit (Tiangen, Beijing, China) following the manufacturer’s instructions. DNA and RNA concentrations were measured using a Qubit^™^ 2.0 Fluorometer (Invitrogen, Carlsbad, CA, United States). 100 ng DNA was used as a template for the construction of a metagenomic high-throughput sequencing library. In the process of library construction, the long DNA fragments were ultrasonically broken by the Covaris M220 instrument (Covaris, Woburn, Massachusetts, USA), and the length of the inserted fragments in the library was about 350 bp. Then, the metagenomic high-throughput library was constructed by using the VAHTS Universal DNA Library Prep Kit for Illumina V3 (Vazyme, Nanjing, China) according to the instructions. 5 μL constructed library was used for 1% agarose gel electrophoresis to verify the library quality. The DNA library was sequenced using an Illumina Novaseq 6000 platform (2×150 bp).

Using the same method, we extracted total RNA from the 11 ISMIFF samples *in-situ* filtered with 0.22 μm pore size membranes. Qubit^™^ 2.0 Fluorescence meter was employed to measure RNA concentration. RNA extracts were treated with DNase I (Tiangen, Beijing, China) as instructed by the manufacturer to ensure DNA removal. PCR was performed to detect DNA residues using the universal primers 341F (5′-CCTAYGGGRBGCASCAG-3′) and 802R (5′-TACNVGGGTA TCTAATCC-3′), and the RNA extracts as a template. Each 50 μL PCR reaction contains 25 μL of 2 × Taq PCR premixed reagent (Tiangen, Beijing, China) and 1 μL of 10 μM primers each. All PCR reaction systems were first heated at 94°C for 3 min, followed by 30 cycles of amplification: 94°C (30 s), 55°C (30 s), and 72°C (1 min), and maintenance at 72°C for 5 min at the end of the cycle. 1% agarose gel electrophoresis was used to confirm that DNA was removed completely. Then, according to instructions for the VAHTS® Universal V8 RNA-seq Library Prep Kit for Illumina (Vazyme, Nanjing, China) kit, the total RNA after DNA removal was used as a template to synthesize double-stranded cDNA and construct a metatranscriptome library. Among them, rRNA was not removed from the total RNA. We then fragmented RNA using conditions of 85°C for 5 min according to the instructions of the reagent kit. 5 μL constructed library was used for 1% agarose gel electrophoresis to verify the library quality. The cDNA library was sequenced using the Illumina Novaseq 6000 platform (2 × 150 bp).

### 2.3 Community composition analysis

Raw Illumina sequencing data of the 21 metagenomes and 11 metatranscriptomes were evaluated using FastQC (v0.11.8).^1^ They were then trimmed to remove adapters and then filtered using fastp (v0.23.1) (Chen et al., 2018) with parameters (-w 24 -c -q 20 -u 20 -g -W 5–3 -l 50). Reads with low quality (assigned by a quality score < 20 for >20% of the read length), those shorter than 50 bp, and unpaired reads were removed. Data with a high level of duplications (>5%) were pre-processed with FastUniq (v1.1) (Xu et al., 2012) to eliminate duplicated reads.

Ribosomal RNA miTags (5.8S, 18S, and 28S) were extracted from the clean metagenomic and metatranscriptomic reads using rna_hmm3.py (Huang et al., 2009), which employed HMMER (v.3.1b2) (Mistry et al., 2013) to predict ribosomal RNA gene fragments from both forward and reverse metagenomic reads. 18S miTags (≥100 bp) were identified using an in-house Python script (Zhou et al., 2022) and matched to the V4 and V9 variable regions of the 18S rRNA gene using HMMsearch software (Eddy, 2011) against V4 and V9 Hidden Markov Models. The 18S miTags were imported into QIIME 2 (v.2022.2) (Bolyen et al., 2019) with the setting of --type “SampleData [Sequences]” and clustered into operational taxonomic units (OTUs) with ≥ 97% similarity using the VSEARCH pipeline (Rognes et al., 2016). The representative miTags were classified using the classify-sklearn command in QIIME 2 in reference to the SILVA SSU 138.1 database (Quast et al., 2013; Glockner et al., 2017). Furthermore, mTAGs (Salazar et al., 2021) were used for additional classification of these miTags, referencing the PR2 (Protist Ribosomal Reference) database (Guillou et al., 2013). After annotation, to account for the differences in sequencing depth between samples, the q2-feature-table command in QIIME2 was used to normalize the feature table, obtaining relative abundance at different taxonomic levels. Three metrics, including Good’s coverage, Chao1, and Shannon index at a 3% dissimilarity were calculated using the QIIME 2 package. The relative abundance of the 18S miTags in 21 metagenomes was imported to the “vegan” package (v.2.5–4) in R to estimate Bray–Curtis dissimilarity index for Principal Coordinate Analysis (PCoA). The dissimilarity of eukaryotic communities in the studied samples of different depths was also analyzed by the unweighted pair group method with arithmetic average (UPGMA) clustering, using the upgma function in the R Vegan package.

### 2.4 Phylogenetic analysis

The reference 18S rRNA gene sequences of Dinophyceae and Maxillopoda were downloaded from the National Center for Biotechnology Information (NCBI) database and the most abundant Dinophyceae and Maxillopoda OTUs in this study were used to construct a phylogenetic tree. The comparison was made using MAFFT (v7.505, setting: –maxiterate 1,000 –localpair) (Katoh and Standley, 2013) and then trimAl (v1.4, setting: -automated1-phylip) (Capella-Gutierrez et al., 2009) was used to construct the alignment results. The maximum likelihood phylogenetic tree of the 18S rRNA genes was constructed using IQ-TREE 2 (v2.2.0, setting: -m MFP -B 1,000 -alrt 1,000 -T AUTO) (Minh et al., 2020). Phylogenetic trees were visualized with iTOL.^2^

## 3 Results

### 3.1 Environmental parameters in sampling stations

**Figure 1:**
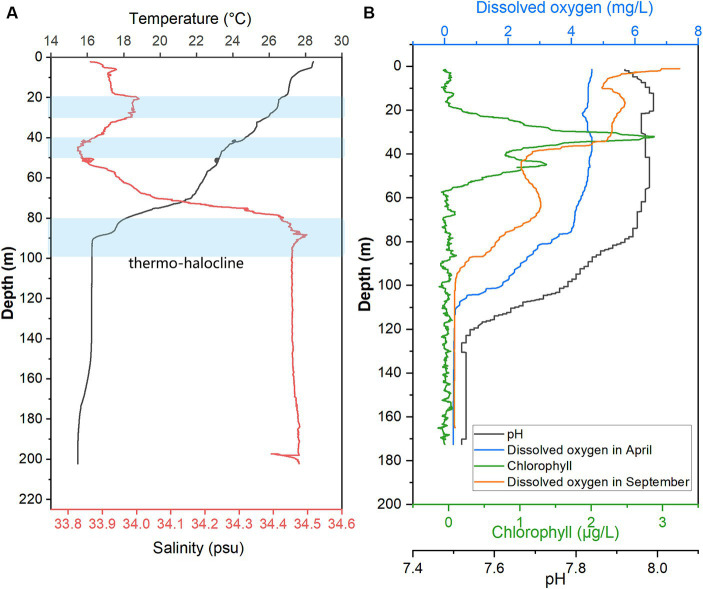
Hydrological parameter profiles in YBH. Profiles of seawater temperature, salinity **(A)**, pH, Chlorophyll, and DO were measured in April and September of 2021 **(B)** inside YBH. The layers with a blue shade represent the thermo- and halocline layers **(A)**.

When sampling from YBH in 2021, we used sensors to measure temperature, pH, salinity, chlorophyll, and dissolved oxygen concentration (DO) in the blue hole (Figure 1). Except for the increased dissolved oxygen concentration in the September cruise, other environmental parameters were detected in the April cruise. The temperature, pH, and dissolved oxygen obtained in this study showed that the readings of these sensors decreased with increasing depth across the water column, and the salinity showed an opposite trend. The temperature decreases with depth from 28.43°C on the surface to 16.30°C at about 90 m and tends to be stable (Figure 1A). The salinity gradually increased from 33.87 at the surface to 34.45 at 95 m and then basically remained stable in the deeper water as previously reported (Xie et al., 2019; He et al., 2020). There are several possible thermo-haloclines due to lower temperatures and fluctuating changes in salinity, and they are located at 20–30 m, 40–50 m, and 80–100 m (Figure 1A). Chlorophyll value peaked at nearly 30 m, reaching 2.88 μg/L. It decreased rapidly with an increment of water depth, and a secondary peak (1.37 μg/L) occurred at ~45 m and then decreased to nearly 0 at ~55 m. Dissolved oxygen concentrations varied seasonally in YBH (Figure 1B). Suboxic layer (0–0.5 mg/L dissolved oxygen) occurred at 105–110 m and 92–101 m in April and September, respectively. Dissolved oxygen in April began to decrease rapidly at about 80 m (~3.37 mg/L), while that in September showed a generally declining trend. Altogether, the YBH water column contained more dissolved oxygen in April than in September. Moreover, in September, YBH had a stronger temperature and redox gradients than in April. Previously, it has been reported that there are multiple thermoclines in YBH, while their depth varies seasonally and the conditions of the anoxic layer are quite stable (Yao et al., 2018; Xie et al., 2019).

During the two cruises, ISMIFF was used to obtain *in-situ* filtration membranes from 16 water layers in a depth range of 1–290 m in April of 2021 (Spring sample), and from five water layers of 96.5–100.5 m and 250 m in September of 2021 (Autumn sample). Among them, the four water layers from 96.5–100.5 m were combined with suboxic samples. A total of 162.48 Gbp and 23.61 Gbp data for metagenomes and metatranscriptomes, respectively, were obtained for all the samples (Supplementary Table S1).

### 3.2 Community composition of eukaryotes in YBH

**Figure 2:**
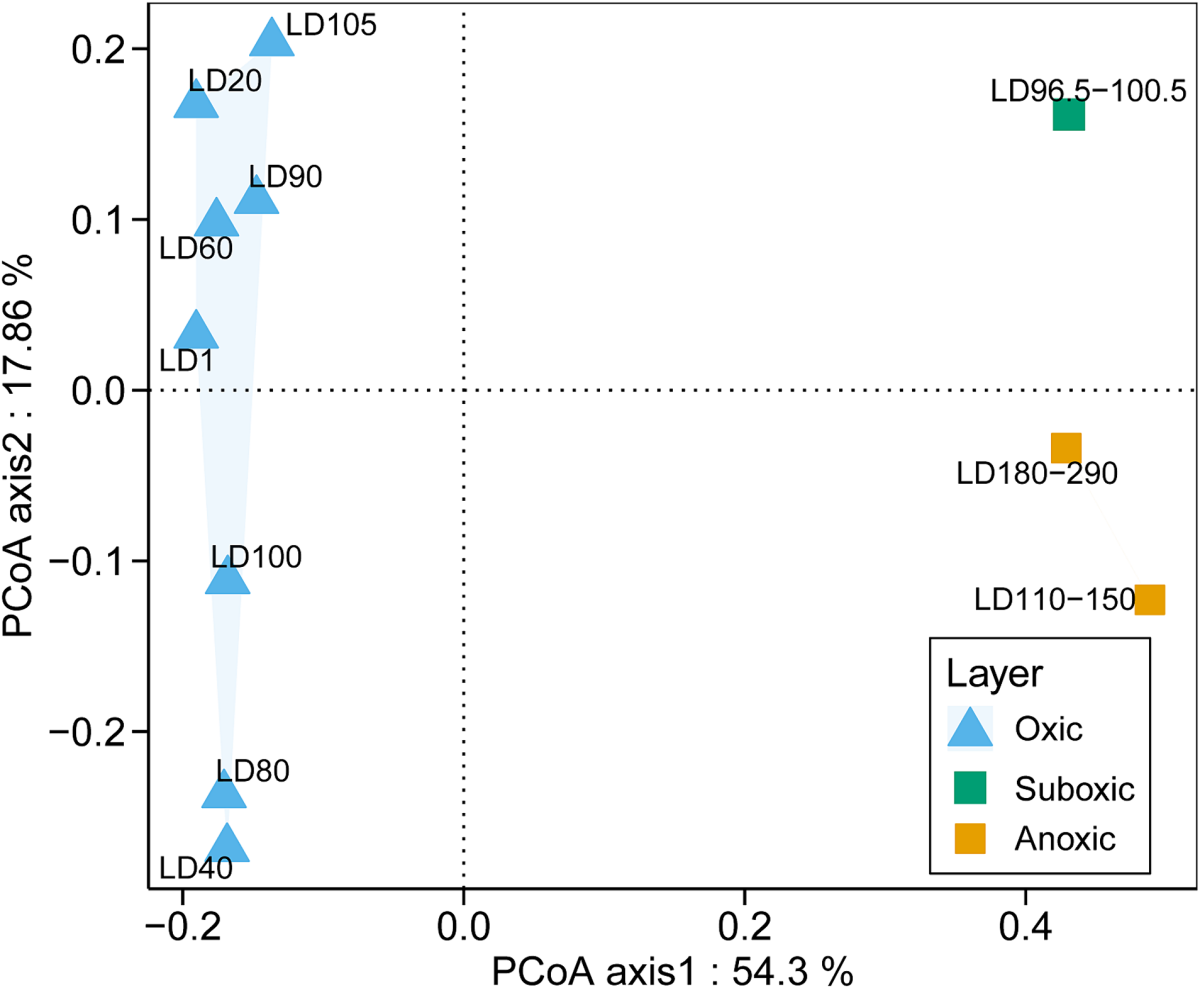
PCoA analysis of eukaryotic community structures. The relative abundances of genera based on the classification of 18S miTags extracted from 21 metagenomes were used for the PCoA analysis. The genera represented by less than 2% in the OTUs were ignored. The sample IDs are referred to Supplementary Table S1.

A total of 16,906 18S miTags assigned for the V4 and V9 regions were extracted from clean Illumina reads of the 21 metagenomes to decipher the eukaryotic microbial communities of YBH. Since the number of 18S V4 and V9 miTags in some samples of the suboxic and anoxic layers was less than 100 (Supplementary Table S1), these 18S miTags of 96.5–100.5 m, 110–150 m, and 180–290 m metagenomes were combined into three individual groups for analysis. To further evaluate the microbial activity, 18S V9 miTags were successfully extracted from clean Illumina reads from 10 metatranscriptomes, which resulted in a total of 381,446 miTags (Supplementary Table S1). The alpha-diversity indices showed that the diversity and richness of the eukaryotic microorganisms in the oxic layer of YBH were significantly higher than those in the suboxic and anoxic layers (U test, *p* < 0.001) (Supplementary Table S1). PCoA plotting showed the divergence of microbial communities at the genus and order levels between different layers (Figure 2; Supplementary Figure S2). The first two principal components accounted for 54.30 and 17.86% of the total variation in the microbial community composition, respectively. In the oxic zone (1–105 m), depth was the main factor driving the division of eukaryotic microbial community structures, explaining 13.35% of the variance in the communities. In contrast, the suboxic (96.5–100.5 m) and anoxic layers (110–290 m) are relatively aggregated, indicating that the community structures resembled each other in the water layers with low oxygen content.

**Figure 3:**
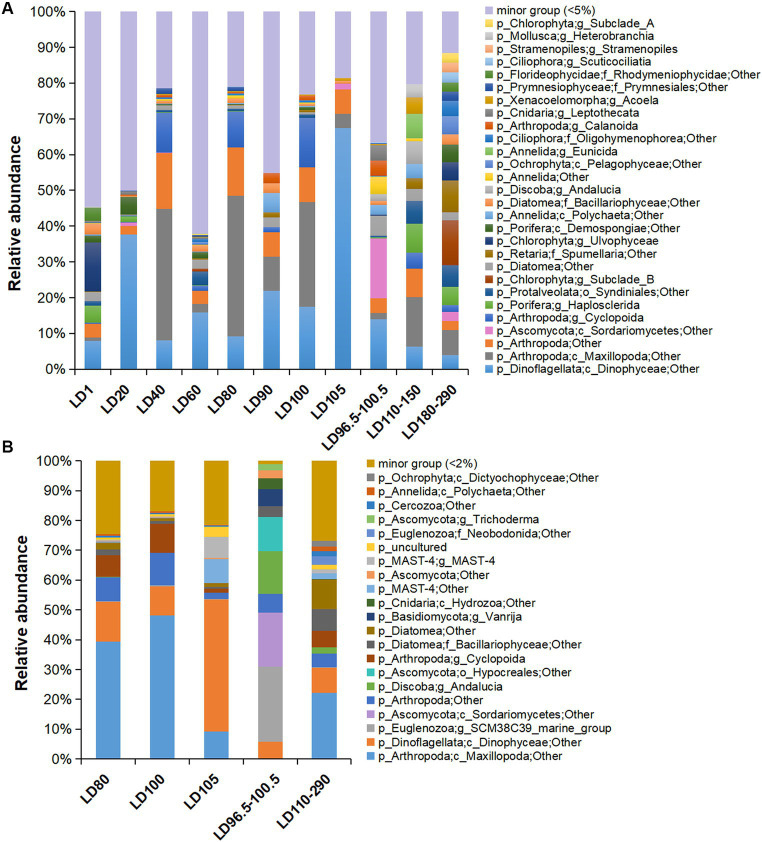
Eukaryotic community structure at genus level based on 18S miTags in metagenomes and metatranscriptomes. **(A)** Eukaryotic community structure in 21 metagenomes. **(B)** Eukaryotic community structure in 10 metatranscriptomes. 18S miTags for the V9 region were extracted from the metagenomes and metatranscriptomes for the generation of OTUs with a 97% similarity. The genera that accounted for less than 5 and 2% of the OTUs for metagenomes and metatranscriptomes, respectively, were grouped into “minor group.” Sampling depth (m) is indicated by the number or number range in the IDs that are described in Supplementary Table S1.

In total, 20 major eukaryotic phyla (relative abundance >5%) including Arthropoda, Dinoflagellata, Chlorophyta, Porifera, and Protalveolata were consistently detected in all the samples, with the removal of unassigned OTUs (~6.34% of all samples) and Eukaryota OTUs that were not classified into phyla (~47.98% of all samples) in the plots (Supplementary Figure S3). The samples were overwhelmed by Arthropoda (~10.49%), followed by Dinoflagellata (~8.41%). The eukaryotes were further divided into 25 major orders (Supplementary Figure S4) and 28 major genera (relative abundance >5%) (Figure 3A). The dominant phyla in the metatranscriptomes were roughly the same as those in the metagenomes, with the exclusion of unassigned OTUs (~2.69%) and Eukaryota OTUs that were not classified into phyla (~41.50%) in the plots (Figure 3B; Supplementary Figure S3B). In addition, we searched the OTUs against the PR2 database, which showed similar results with the classification based on the Silva database (Supplementary Figure S5). Additionally, we showed the structure of the protozoan community after the removal of the metazoans (Arthropoda, Porifera, Annelida, Cnidaria, Xenacoelomorpha, and Mollusca), in which Dinoflagellata was overwhelmingly dominant (~39.23% of all samples) (Supplementary Figure S6). However, copepod groups have been found to be in higher abundance in the hole (Chen et al., 2018), and 18S rRNA gene amplicon data have been used to assess the metazoan diversity in the pico-nanoplanktonic samples (López-Escardó et al., 2018). Therefore, in order to comprehensively understand the diversity of eukaryotic microorganisms, subsequent analyses included data that encompassed metazoan.

The presence of short 18S miTags in this study may have contributed to the potential inaccuracy of eukaryotic taxonomic classification. To address this, we compared the 18S V4 miTags with V9 miTags in the consistency of eukaryotic microbial community structures. Our results obtained using the V4 miTags of the 18S rRNA genes (Supplementary Figure S7) were similar to those based on the V9 ones. Both regions demonstrated the same dominant taxa (Dinophyceae and Maxillopoda). The hypervariable V9 region has been used to independently evaluate eukaryotic microbial diversity at the population level with the availability of a good reference database (Obiol et al., 2020).

Maxillopoda (Arthropoda) were dominant in 40 m (22.84%), 80 m (23.19%), and 100 m (15.42%) water depths, respectively. In the anoxic zone of this study, a certain abundance of Maxillopoda (4.62%) was identified in the metagenomes and was transcriptionally active (about 10.66% of the metatranscriptome-based communities) (Figure 3B). Dinophyceae was the main group of eukaryotic algae with the highest abundance in 20-m (20.01%) and 105-m (26.13%) depth metagenomes, and dominated the transcriptomes from 105 m depth (15.35%) (Figure 3B). The class Sordariomycetes (Ascomycota) prevailed at 98.5 m depth, as they accounted for 16.74% of the metagenome and 18.12% of the transcriptome of the suboxic samples (Figure 3B). Similar to the surface layer, Chlorophyta (11.59%) and Porifera (6.54%) were also rich in the deep anoxic layers (180–290 m depths) (Figure 3A). Interestingly, Porifera showed transcriptional activity at 290 m depth (genus *Haplosclerida*, occupying about 0.06% of the transcribed 18S miTag V9 reads), and their distribution is currently known to be restricted in shallow water (Redmond et al., 2011). Moreover, the taxonomic status of the Porifera remains to be determined.

**Figure 4:**
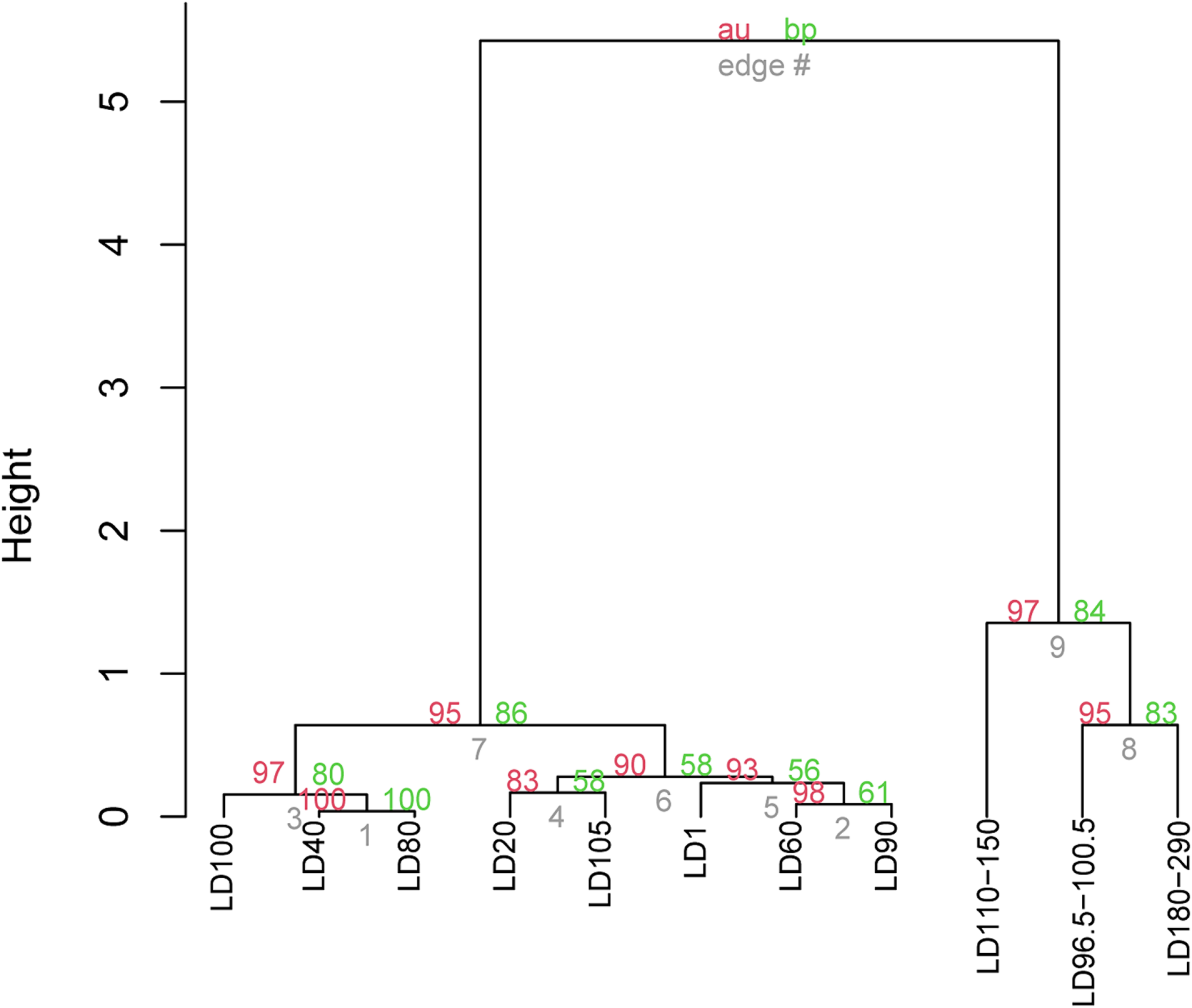
UPGMA clustering of the eukaryote communities in YBH. The clustering was based on the relative abundance of eukaryote communities at genus level in 21 metagenomes. The genera represented by less than 2% in the OTUs were ignored. The *p*-values (%) were calculated using the pvclust function, where the red number represents the AU (approximately unbiased) *p* value of the cluster, and the green number represents the BP (bootstrap probability) of the cluster. The sample IDs are referred to Supplementary Table S1.

UPGMA clustering was applied to compare the community similarity and dissimilarity of different depths based on metagenomic 18S V9 miTags. Our result showed that the samples from different oxygenated layers could be divided into two clades, the first consisting of those sampled from 40 m, 80 m, and 100 m depths in April of 2021 and the second comprising the rest (Figure 4). Compared with suboxic and anoxic layers, the community structures of the oxic layers were more similar. It suggests that oxygen availability plays a significant role in shaping eukaryotic community structures, and the eukaryotic communities in the oxic layers exhibit similar composition patterns. At the sampling depths (40 m, 80 m, and 100 m) with a boundary of thermo-halocline (Figure 1A), the eukaryotic communities resembled the prevalence of Arthropoda in these layers, probably accounted for the thermo-halocline and the oxic-anoxic interface that hinder the vertical movement of eukaryotes (Lovejoy et al., 2006; Weber et al., 2014; Edgcomb, 2016).

### 3.3 Phylogenomics of Dinophyceae and Maxillopoda

**Figure 5:**
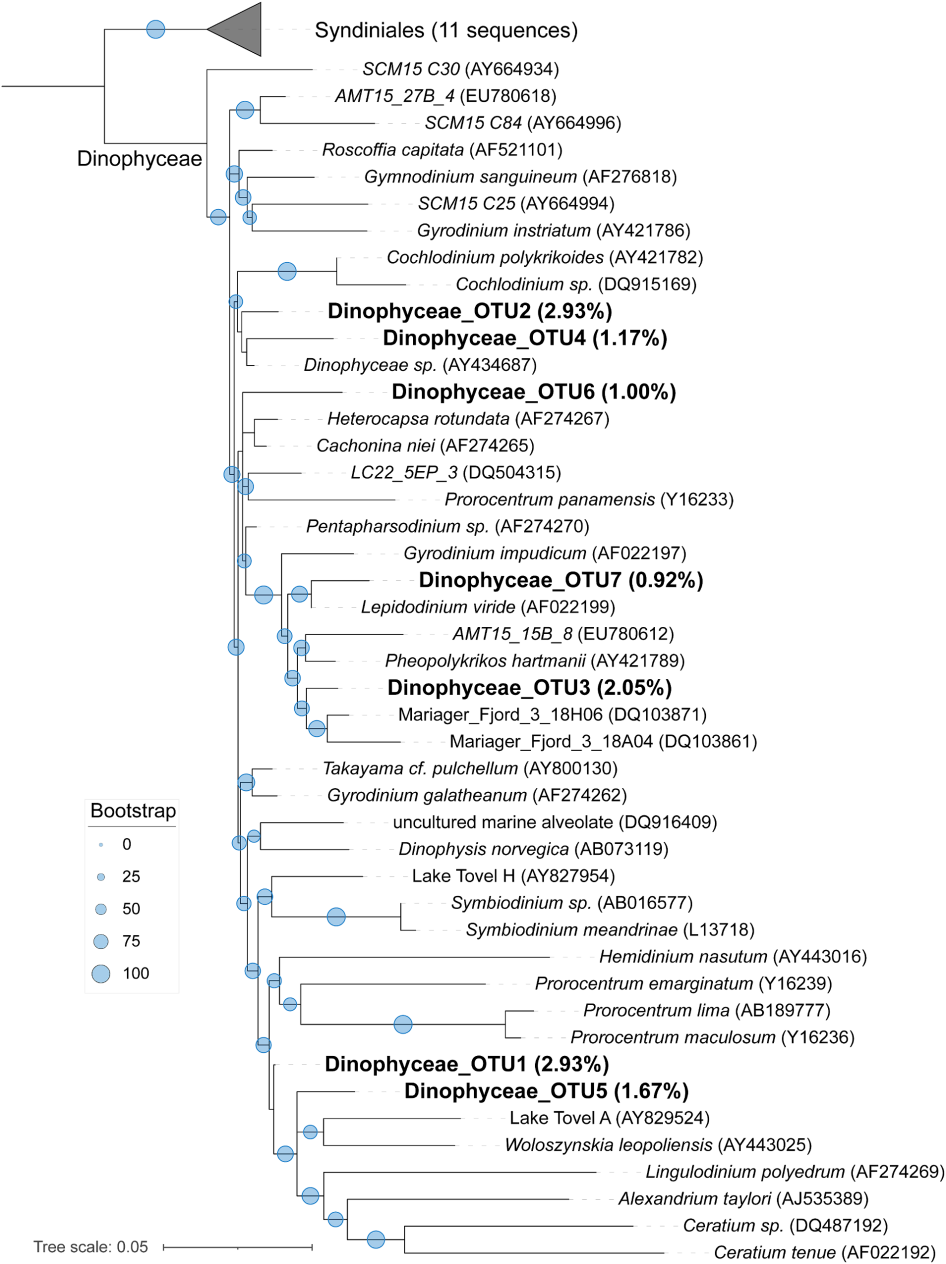
Maximum-likelihood phylogenetic tree of dominant OTUs of Dinophyceae class in YBH samples. The representative sequences of the dominant OTUs based on 18S V9 miTags were aligned with the closest references from NCBI for the construction of the ML tree. Bootstrap values above 50 based on 1,000 replicates are shown as dots with different sizes. The highest relative abundance of these OTUs in themetagenomes for 20-m and 105-m depths is indicated as a percentage in the parentheses.

**Figure 6:**
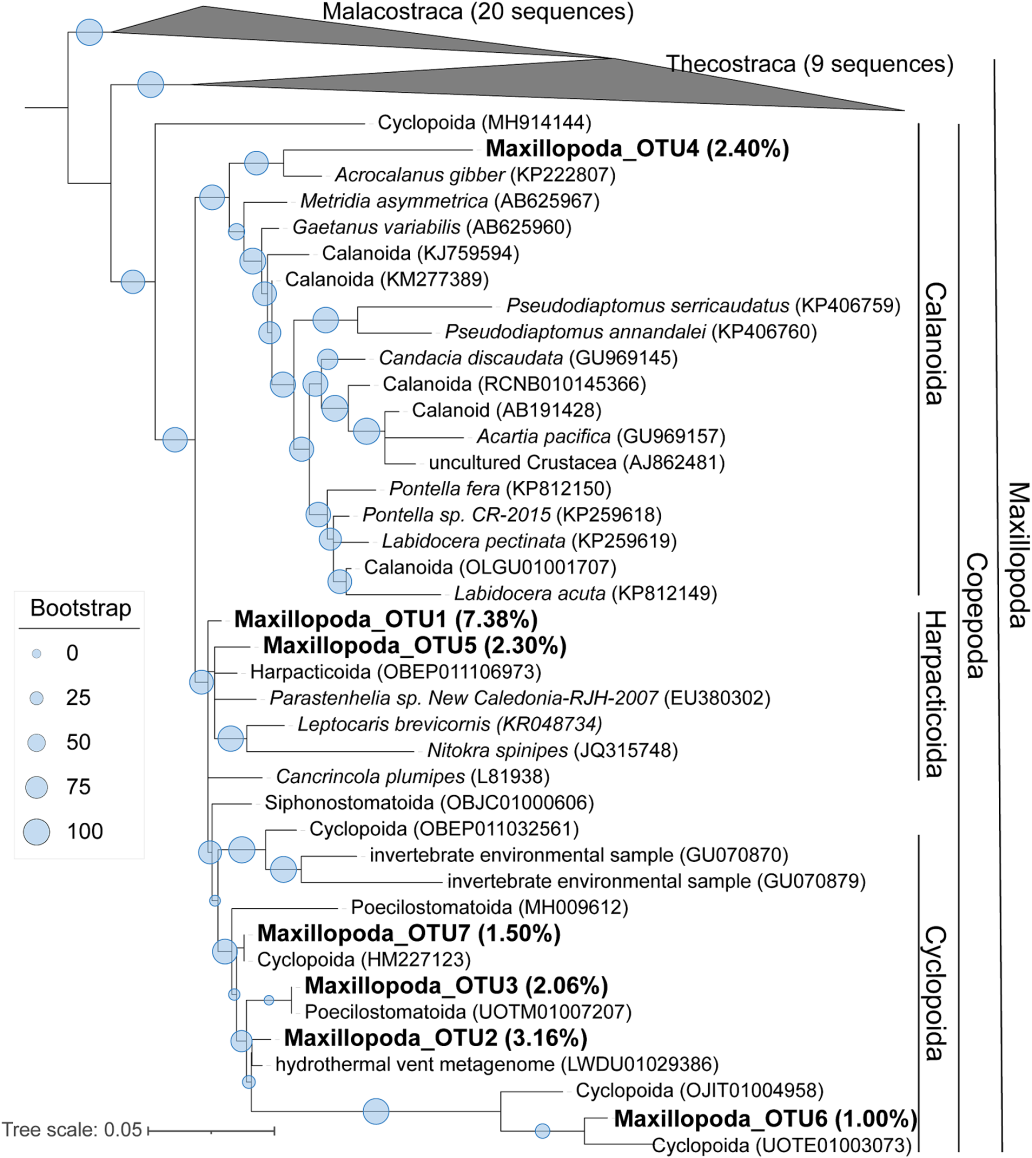
Maximum-likelihood phylogenetic tree of dominant OTUs of Maxillopoda class in YBH samples. The representative sequences of the dominant OTUs based on 18S V9 miTags were aligned with the closest references from NCBI for the construction of ML tree. Bootstrap values above 50 based on 1,000 replicates are shown as dots with different sizes. The highest relative abundance of these OTUs in the metagenomes for 40-m, 80-m, and 100-m depths is indicated as a percentage in the parentheses.

To learn a more detailed taxonomic affiliation of the most abundant Dinophyceae OTUs of 18S V9 miTags, we reconstructed phylogenetic trees using their representative joint reads from the 20 m and 105 m metagenomes, respectively (Figure 5). The reference sequences of the cultured strains were selected from the NCBI database. These Dinophyceae OTUs were almost all adjacent to known strains with high bootstrap values except for OTU1, OTU2, and OTU4 which occupied, on average, 2.93, 2.93, and 1.17% of the metagenome-based communities at 20 m and 105 m depths. These OTUs (0.92% ~ 2.93%) were scattered into different families of Dinophyceae. Syndiniales group I has been found to survive in anoxic and suboxic ecosystems (Guillou et al., 2010), and the OTUs from this study were not assigned to Syndiniales. Similarly, the most abundant Maxillopoda OTUs showed a phylogenetical affinity with the references from subclass Copepoda that were prevalent in YBH (Chen et al., 2018), indicating that this taxon is a dominant, highly active member in the oxic zone. Although most of the OTUs could be found in an adjacent position of *Calanoida*, *Harpacticoida,* and *Cyclopoida* in the phylogenetic tree (Figure 6), OTU2 (~3.16%) was aggregated with uncultured species. In our phylogenetic tree, Arthropoda OTU1 (~7.38%), OTU3 (~2.06%), and OTU5 (~2.30%) were proximate to the orders from Harpacticoida and Poecilostomatoida.

## 4 Discussion

In the present study, we investigated the stratified distribution and diversity of eukaryotic microbes in the water column of YBH using metagenomes and metatranscriptomes. It was observed that plankton in the cave exhibited diurnal vertical movement, particularly in the water layer above 90 m. This area has a strong thermo-halocline that may have hindered the zooplankton’s vertical migration (Chen et al., 2018), possibly explaining the higher abundance of arthropods at the depths of 80 m and 100 m compared to 90 m. The Arthropoda phylum showed high transcriptional activity at depths of 110–290 m (15.79%), indicating the presence of arthropods in the anoxic layer. Copepods with high abundance have been observed through microscopy (Chen et al., 2018), supporting the existence of Maxillopoda in the anoxic zone. However, it has been reported that the oligotrophic condition in the deep anoxic environment of the Cariaco Basin limits the survival rate of arthropods (Stoeck et al., 2003). Copepoda is a group of small Arthropoda with prosome adult length less than 2 mm (Roura et al., 2018). The smallest Copepoda is about 20 μm in size (Steinberg and Landry, 2017). Previous studies on Copepoda microbes from the ocean used 64-μm and even larger mesh nets (Shoemaker et al., 2019; Barth-Jensen et al., 2022; Sugai et al., 2023). In this study, we used two sets of filtration membranes to capture Arthropoda in sizes of 0.22–30 μm. However, from our knowledge, the adults of Harpacticoida and Poecilostomatoida were mostly larger than 30 μm (El-Rashidy and Boxshall, 1999; Dahms and Qian, 2004). Therefore, we believe that some of the Arthropoda captured were larvae harvesting bacterial preys in YBH. Furthermore, we cannot exclude the possibility of encountering novel Arthropoda species smaller than 30 μm in their adult stage. They were likely attracted by swarming chemosynthetic bacterial preys prevailing at the thermo-halocline boundaries and oxic-anoxic interface (Chen et al., 2018).

The growth of eukaryotic algae is affected by light intensity. However, excessive light can inhibit algae growth, as previously reported (Takahashi et al., 1973; Liu et al., 2019). The algae bloom at 20 m depth might be related to optimal light intensity in this study. The higher transcriptional activity of Dinophyceae may indicate an unknown branch capable of growing in lightless and low-oxygen environments. Dinophyceae that have been reported were largely found in the photic zone (Luo et al., 2022). Dinoflagellates are believed to be capable of phagotrophic nutrition (Sherr et al., 2007). Most of the Dinophyceae sequences were closely related to known cultured representatives or environmental clones from other hypoxic marine systems (Mariager Fjord) and an oligotrophic mountain lake (Lake Tovel) (Di Giuseppe and Dini, 2004; Zuendorf et al., 2006). Further genomic and transcriptomic studies of these OTUs will provide evidence for their metabolic activities in YBH.

The marine fungus Sordariomycetes is commonly found in marine sediments and is often associated with corals and sponges or parasitic for algae (Jones et al., 2019). Its prevalence at a depth of 98.5 m might be due to the abundance of organic particles for degradation or its parasitism with eukaryotic algae. Chlorophyta, a class of photosynthetic green algae, was classified as class Clade_VII and Ulvophyceae, which are typically found in euphotic environments (Hodgson and McDermid, 2000). However, their low abundance in the metatranscriptomes of deep anoxic layers (180–290 m) suggests that they may be less active in these conditions. Further investigation is needed to determine whether the Chlorophyta were permanent inhabitants of the YBH’s deep layer. The SCM38C39 marine group (phylum Euglenozoa, order Diplonemea) was found in the 100.5 m suboxic layer. While it accounted for a small percentage of the community (<2%) based on the metagenomics reads, its high activity (18.45%) in based on the metatranscriptomics reads. Diplonemea, known for preying on bacteria, is one of the most abundant groups of marine planktonic protists (Flegontova et al., 2020). It possesses enzymes for anaerobic adaptation (NADP^+^ oxidoreductase, fumarate reductase, enoyl- coenzyme A reductase, wax ester synthesis enzymes), which enables it to thrive in low-oxygen environments (Škodová-Sveráková et al., 2021). Diplonemea have been found in the anoxic Cariaco Basin (Schoenle et al., 2021)and might be more active in suboxic zones compared to other protists, as indicated by high transcriptomic levels.

Eukaryotic microalgae, specifically the Dinophyceae, dominated the water column at depths of 20 m and 105 m, and they might contribute significantly to carbon input in the YBH ecosystem. These microorganisms, along with Cyanobacteria on the surface, produce organic carbon, which provides important energy for the entire food web (He et al., 2020; Yao et al., 2020). Eukaryotic microorganisms, including Dinophyceae and Maxillopoda, play a role in nutrient cycling within the YBH. The presence of swarming chemosynthetic bacterial prey at the thermo-halocline boundaries and oxic-anoxic interface attracts larvae and protists, including Maxillopoda. These eukaryotic microorganisms likely feed on the bacterial preys (Pernthaler, 2005), contributing to the transfer of energy and nutrients within the ecosystem. Additionally, Maxillopoda may serve as a food source for higher trophic levels, such as fish and larger invertebrates (Suárez-Morales, 2015). The presence of different species at specific depths suggests that the blue hole provides distinct ecological niches for these organisms. This diversity of eukaryotes contributes to the overall biodiversity of the ecosystem and can influence species interactions, such as competition and predation, which shape the structure and dynamics of the community.

Other blue holes and typical anoxic water columns have similar sulfided environments such as that in the Black Sea (Edgcomb et al., 2014; Cabello-Yeves et al., 2021). There are abundant ciliates and flagellate taxa (mainly stramenopiles and dinoflagellates) in anoxic, especially sulfidic water layers, and the species diversity in sulfide layers is higher than that in hypoxic samples (Wylezich and Jürgens, 2011). By contrast, the main dominant phyla of the suboxic and anoxic sulfide water column in YBH are Arthropoda and Dinoflagellata as shown by this study. In YBH, the anoxic layer had lower species diversity compared to the suboxic layer. In the anoxic layer of YBH, ciliates mainly existed at 290 m depth (6.06%), belonging to the class Oligohymenophorea. However, the genus *Cyclidium* (Oligohymenophorea) which is the dominant bacterial group in the sulfidic layer of the Black Sea, may be a potential bacterial consumer and preys on sulfur-reducing bacteria. Compared with the Black Sea, ciliates are not eukaryotic microorganisms that dominate YBH. In summary, the eukaryotic microorganisms found in the anoxic and sulfided environment of YBH provide new evidence for the adaptation and evolution of early eukaryotes to anoxic habitats (Porter et al., 2018).

There are a large number of eukaryotes that have not been classified into phyla using current references in public databases, which can indicate that there are more novel species in YBH. We suggest more efforts for the exploration of the anaerobic eukaryotic microbes in the anoxic layer of YBH. In addition, although it is a fact that the size of Maxillopoda in general is very small, they are multicellular organisms. Their dominance in the present study might be caused by having multiple copies of the rRNA gene in their genome (Hu et al., 2016; Ibarbalz et al., 2019). However, their high abundance has been identified through morphological observation, a method that is beyond doubt for confirming their presence (Chen et al., 2018). Moreover, we also filtered the samples from over 30 L to avoid sampling drift. Nevertheless, this is an unavoidable limitation of the metagenomics method for marker gene analysis. Since we only analyzed samples <30-μm and included only protists and some larval communities of large eukaryotes (e.g., Maxillopoda, etc.), there was a lack of larger eukaryotes to show the dependence between prokaryotes and eukaryotes in the water column of the hole. Additional analysis will be conducted on eukaryotic communities within samples larger than 30-μm, in order to achieve a more systematic and comprehensive examination of the distinct ecosystem within the YBH.

## 5 Conclusion

This study analyzed the eukaryotic communities in various chemical gradient layers of YBH using metagenomics and metatranscriptomics and showed the dominant position of Dinophyceae and Maxillopoda in different layers. Dinophyceae were the most abundant at the 20 m and 105 m water depths, while Maxillopoda were dominant at the 40 m, 80 m, and 100 m. Some of these organisms were likely larvae and protists attracted by the chemosynthetic bacterial prey at the thermo-halocline boundaries and oxic-anoxic interface. The environmental barrier of protozoa in the hole hinders the vertical movement of protozoa and forms a stable ecological niche, which provides a microbial oasis for the stability of the material cycle in the hole. The phylogenetic relationships of the major 18S OTUs were mostly adjacent to known species, except for three Dinophyceae OTUs, pointing to the potential taxonomic novelty of these organisms. Consequently, our study has provided a detailed look at the vertical distribution of <30-μm eukaryotic microbes in the YBH, underscoring the taxonomic novelty and ecological importance of eukaryotic microorganisms in these unique semi-enclosed blue holes. The YBH hosts numerous endemic species that require further studied in terms of taxonomy and metabolic activities. In summary, this study presents important evidence for existence of eukaryotic microorganisms in suboxic and anoxic layers of marine blue hole ecosystems. Future efforts may further explore the metabolic pathways, energy acquisition strategies, and material cycling mechanisms of these eukaryotic microorganisms. This will ultimately provide in-depth understanding of the adaptability, diversity, and origin of eukaryotic organisms.

## Data availability statement

18S miTags of YBH are available at the NCBI Sequence Read Archive (SRA) under BioProject PRJNA900714 (BioSample accession numbers SAMN31699490–SAMN31699510).

## Author contributions

HZ: Conceptualization, Formal analysis, Visualization, Writing – original draft. TW: Formal analysis, Resources, Writing – review & editing. QL: Formal analysis, Resources, Writing – review & editing. LF: Resources, Writing – review & editing. ML: Writing – review & editing. LH: Resources, Writing – review & editing. YW: Conceptualization, Writing – review & editing.

## Footnotes

^1^http://www.bioinformatics.babraham.ac.uk/projects/fastqc/

^2^https://itol.embl.de/
