# Supplementary material for: Metagenomic 18S rDNA reads revealed zonation of eukaryotic communities in the Yongle blue hole
Source: Front Microbiol. 2024 Jul 29;15:1420899. doi: 10.3389/fmicb.2024.1420899 (PMC11317397; doi:10.3389/fmicb.2024.1420899)
Supplement: Supplementary file 1 [file Table_1.DOCX]

Supplementary Material

# Supplementary Figures and Tables

## Supplementary Tables

**Table S1** Information of sampling sites and statistics of metagenomic sequencing data and 18S miTags.

| **Depth** | **Sample ID** | **Region** | **Collection date** | **Metagenome** | | **Metatranscriptome** | |
| --- | --- | --- | --- | --- | --- | --- | --- |
|  |  |  |  | **Total bases (Gbp)** | **No. 18S miTags^a^** | **Total bases (Mbp)** | **No. 18S miTags** |
| 1m | LD1 | Oxic layer | 2021/4/25 | 2.20 | 950 |  |  |
| 20m | LD20 | Oxic layer | 2021/4/24 | 2.30 | 1197 |  |  |
| 40m | LD40 | Oxic layer | 2021/4/24 | 2.81 | 6556 |  |  |
| 60m | LD60 | Oxic layer | 2021/4/24 | 11.53 | 2111 |  |  |
| 80m | LD80 | Oxic layer | 2021/4/24 | 5.44 | 2347 | 288 | 2307 |
| 90m | LD90 | Oxic layer | 2021/4/24 | 3.04 | 185 |  |  |
| 100m | LD100 | Oxic layer | 2021/4/24 | 5.10 | 605 | 9289 | 354699 |
| 105m | LD105 | Oxic layer | 2021/4/26 | 5.06 | 342 | 5671 | 22642 |
| 96.5m | LD96.5 | Suboxic layer | 2021/9/17 | 36.16 | 355 | 11 | 0 |
| 98.5m | LD98.5 | Suboxic layer | 2021/9/17 | 4.39 | 1722 | 356 | 10 |
| 99.5m | LD99.5 | Suboxic layer | 2021/9/16 | 26.56 | 68 | 7 | 251 |
| 100.5m | LD100.5 | Suboxic layer | 2021/9/16 | 5.63 | 30 | 195 | 16 |
| 110m | LD110 | Anoxic layer | 2021/4/24 | 9.16 | 138 | 5113 | 75 |
| 120m | LD120 | Anoxic layer | 2021/4/26 | 16.39 | 74 |  |  |
| 130m | LD130 | Anoxic layer | 2021/4/26 | 2.85 | 11 | 2953 | 1264 |
| 140m | LD140 | Anoxic layer | 2021/4/25 | 3.25 | 47 |  |  |
| 150m | LD150 | Anoxic layer | 2021/4/25 | 4.12 | 23 |  |  |
| 180m | LD180 | Anoxic layer | 2021/4/25 | 3.60 | 18 |  |  |
| 200m | LD200 | Anoxic layer | 2021/4/26 | 4.14 | 26 |  |  |
| 250m | LD250 | Anoxic layer | 2021/9/16 | 5.15 | 63 | 48 | 1 |
| 290m | LD290 | Anoxic layer | 2021/4/26 | 3.62 | 38 | 240 | 181 |

^a^ The 18S rRNA gene reads from the Illumina metagenomic sequences (miTags) were extracted for taxonomic classification. Some miTags were located in the V9 region of the 18S rRNA genes, while the others were derived from all the regions.

## Supplementary Figures


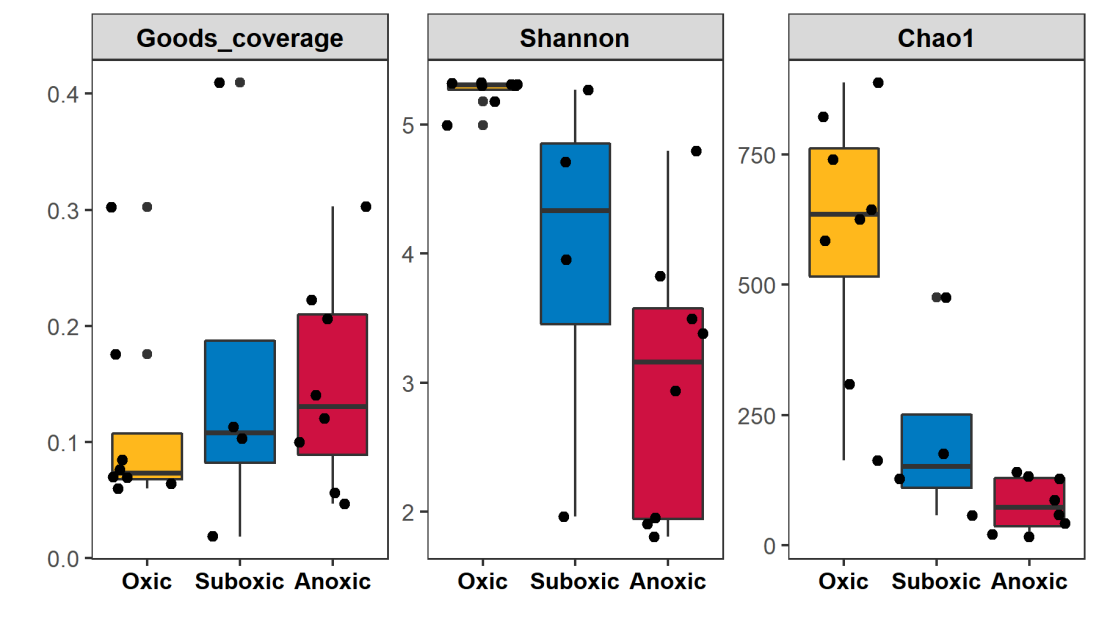


**Supplementary Figure 1** Microbial alpha diversity indices of the eukaryotic microbial communities based on 18S V9 miTags of 21 metagenomes. The Good’s coverage, Chao1, and Shannon index were calculated at a 3% dissimilarity using metagenomic 18S miTags from the V9 region.


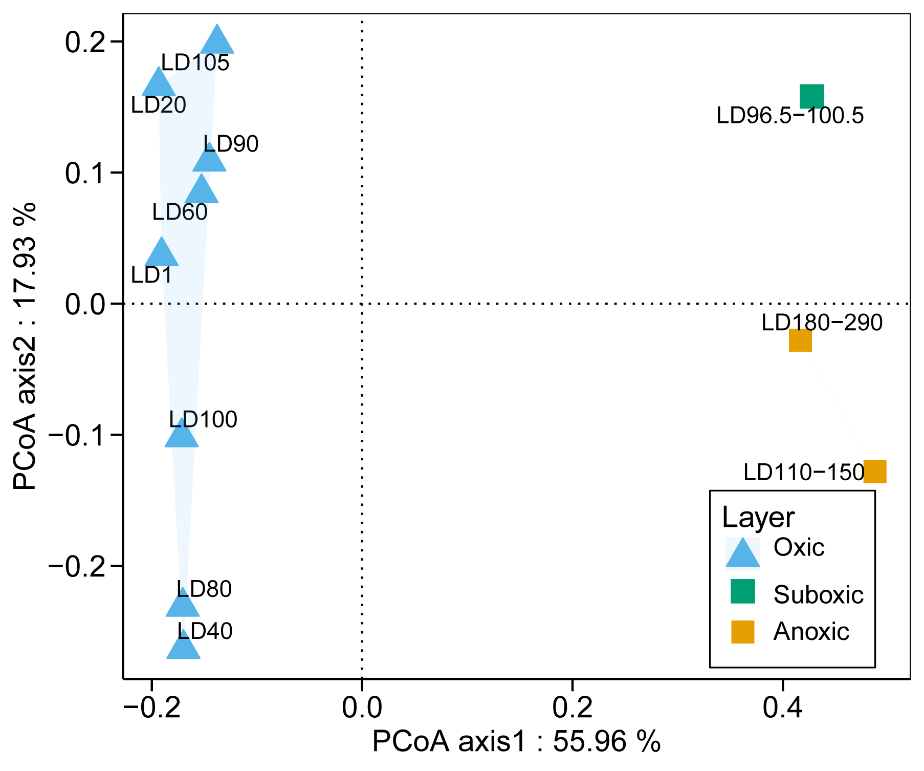


**Supplementary Figure 2** PCoA analysis of eukaryotic community structures. The relative abundances of orders based on the classification of 18S V9 miTags extracted from 21 metagenomes were used for the PCoA analysis. The orders represented by less than 2% in the OTUs were ignored. The sample IDs are referred to [Table S1](https://www.sciencedirect.com/science/article/pii/S0967063718303133#t0005).


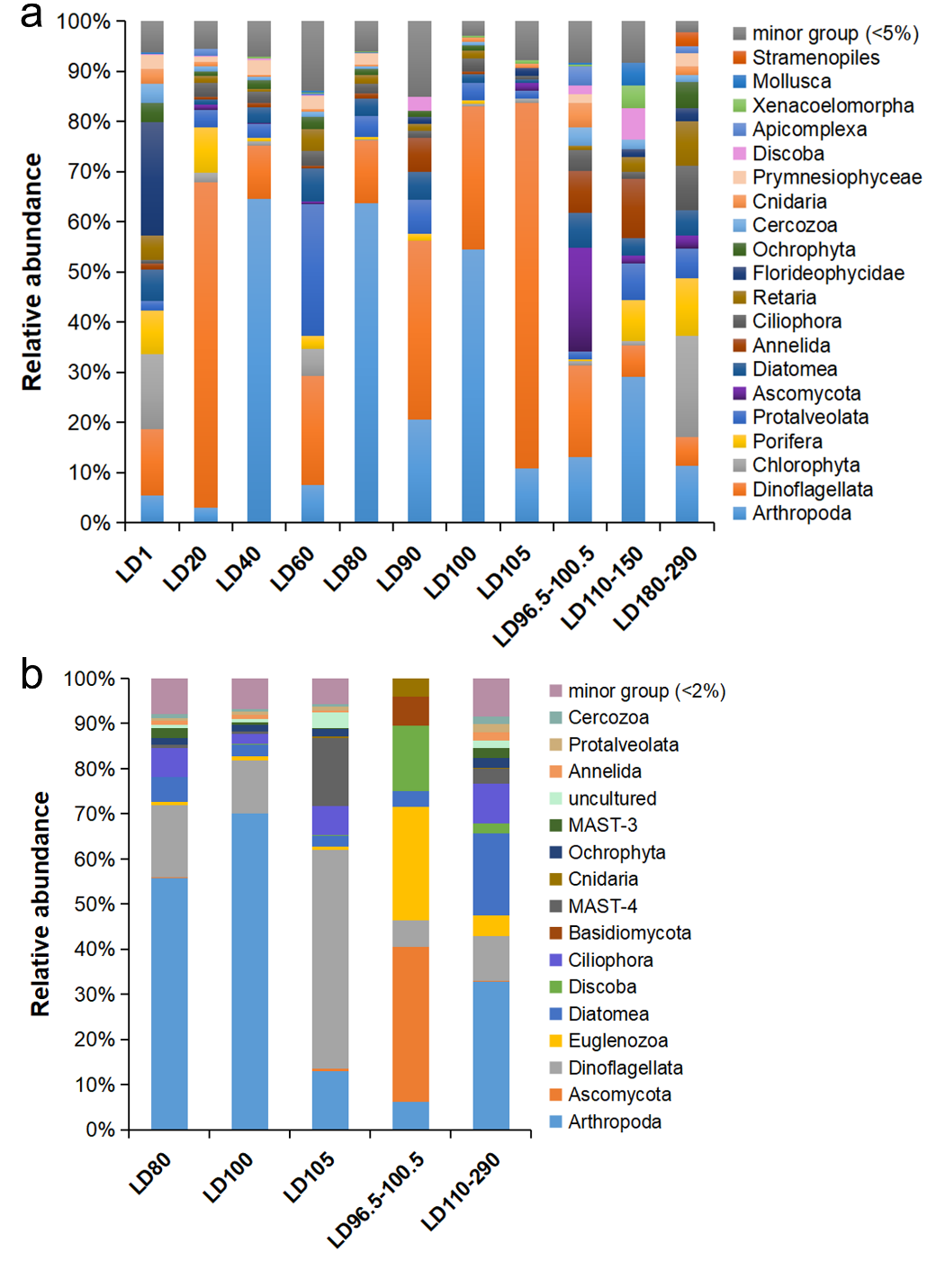


**Supplementary Figure 3** Eukaryotic community structures based on 18S V9 miTags from 21 metagenomes and 10 metatranscriptomes at phylum level. a, Eukaryotic community structure based on the miTags from 21 metagenomes. b, Eukaryotic community structure based on the miTags from 10 metatranscriptomes. The phyla that accounted for less than 5% and 2% of the OTUs for metagenomes and metatranscriptomes respectively were grouped into “minor group”. Sampling depth (m) is indicated by the number or number range in the IDs that are described in Table S1.


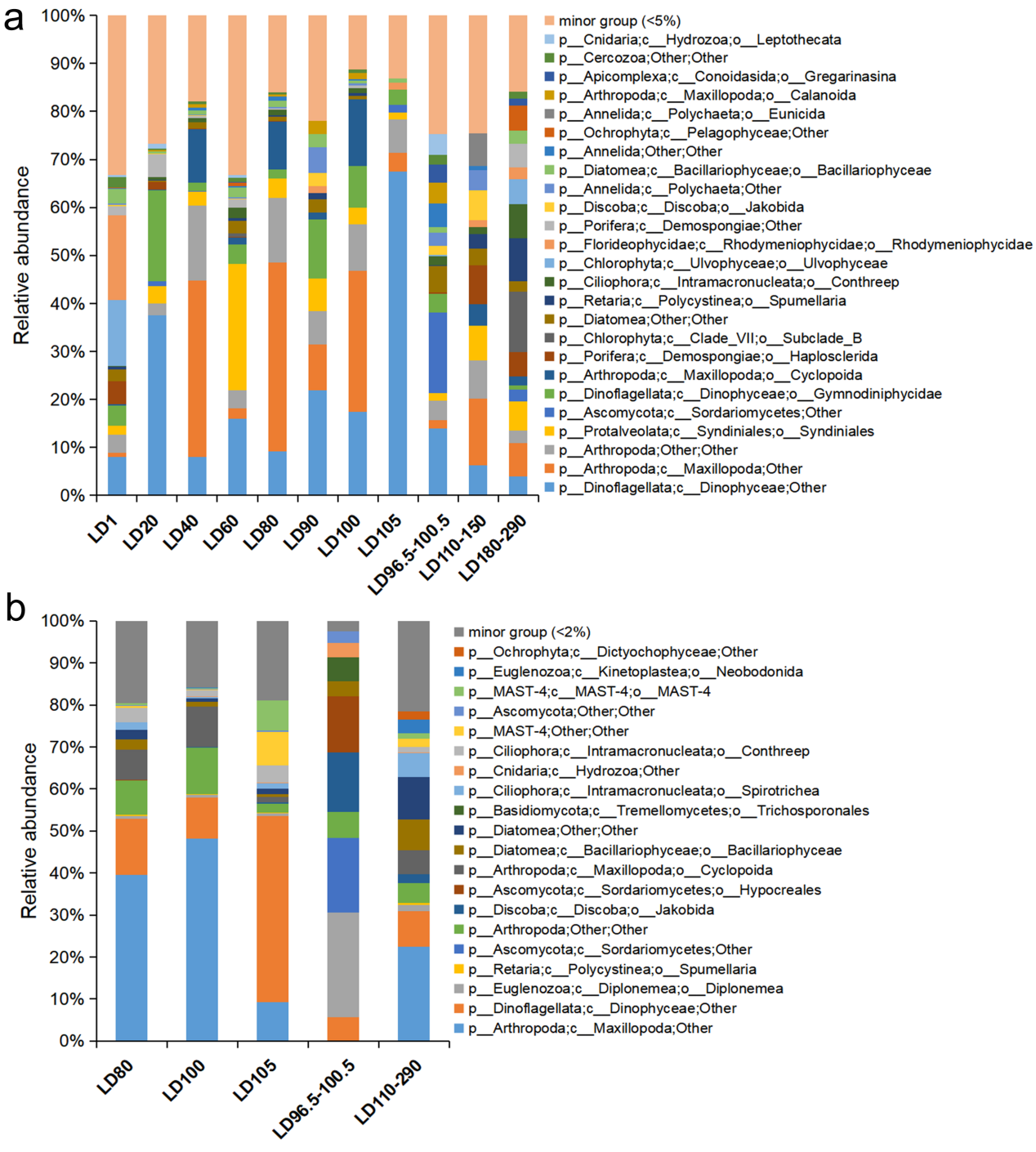


**Supplementary Figure 4** Eukaryotic community structures based on the 18S V9 miTags from 21 metagenomes and 10 metatranscriptomes at order level. a, Eukaryotic community structures based on the 18S V9 miTags from 21 metagenomes. b, Eukaryotic community structures based on the 18S V9 miTags from 10 metatranscriptomes. The genera that accounted for less than 5% and 2% of the OTUs for metagenomes and metatranscriptomes respectively were grouped into “minor group”. Sampling depth (m) is indicated by the number or number range in the IDs that are described in Table S1.


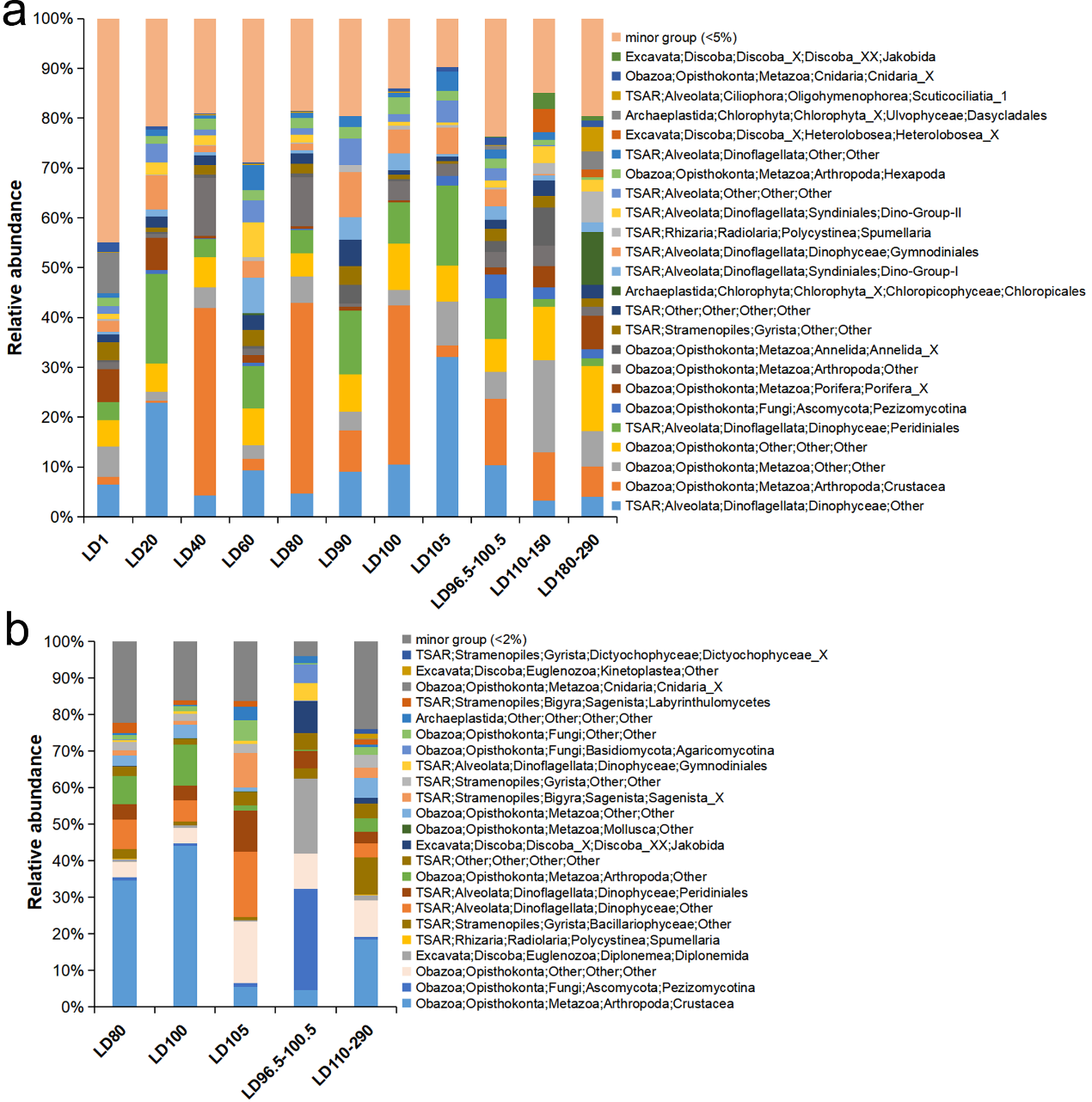

**Supplementary Figure 5** Eukaryotic community structures based on the 18S V9 miTags from 21 metagenomes and 10 metatranscriptomes at order level in reference to PR2 database. a, Eukaryotic community structures based on the 18S V9 miTags from 21 metagenomes. b, Eukaryotic community structures based on the 18S V9 miTags from 10 metatranscriptomes. The genera that accounted for less than 5% and 2% of the OTUs for metagenomes and metatranscriptomes respectively were grouped into “minor group”. Sampling depth (m) is indicated by the number or number range in the IDs that are described in Table S1.


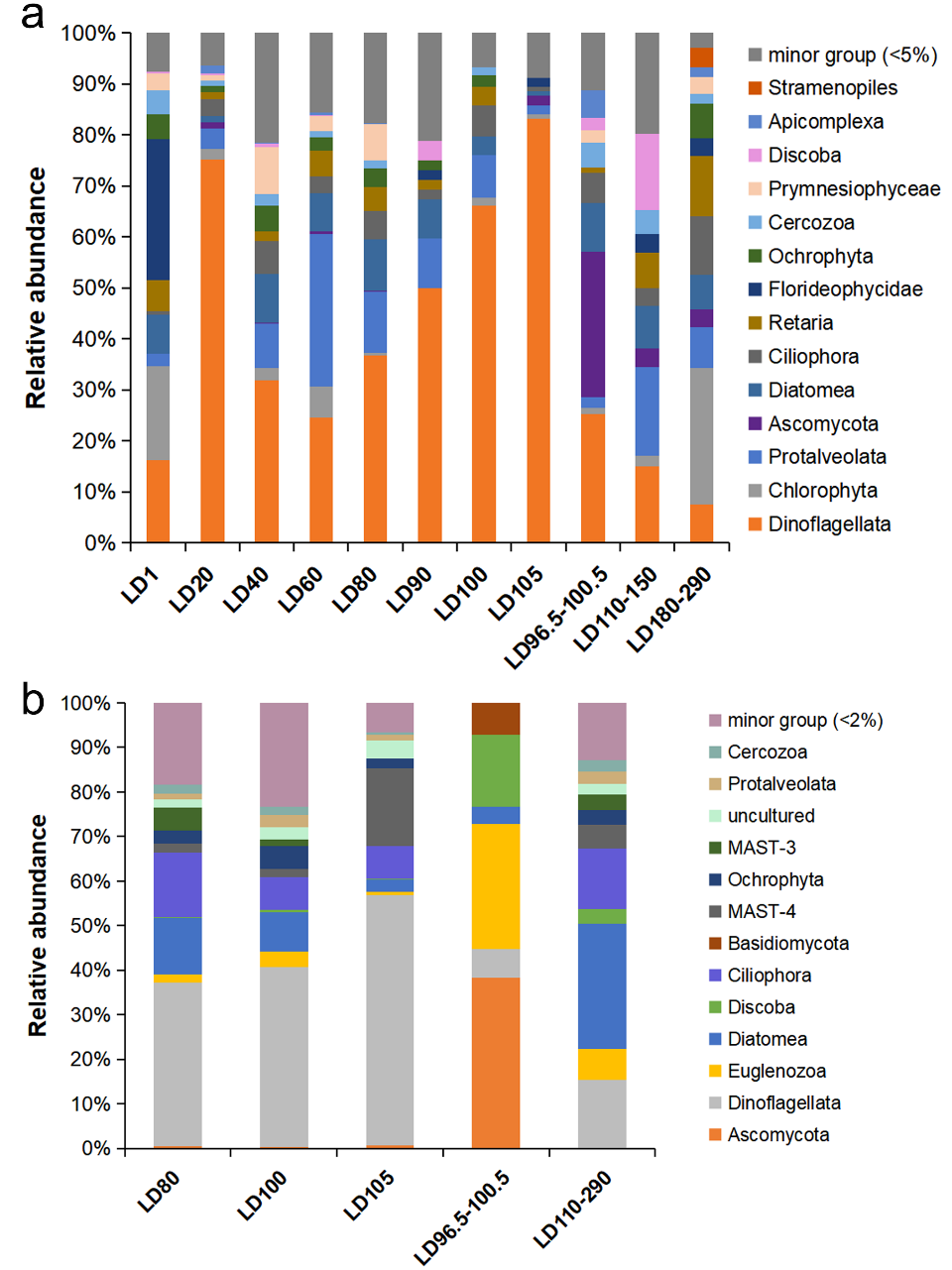


**Supplementary Figure 6** Protist community structures based on the 18S V9 miTags from 21 metagenomes and 11 metatranscriptomes at phylum level. a, Protist community structures based on the 18S V9 miTags from 21 metagenomes. b, Protist community structures based on the 18S V9 miTags from 10 metatranscriptomes. The phyla that accounted for less than 5% and 2% of the OTUs for metagenomes and metatranscriptomes, respectively, were grouped into “minor group”. Sampling depth (m) is indicated by the number or number range in the IDs that are described in Table S1.


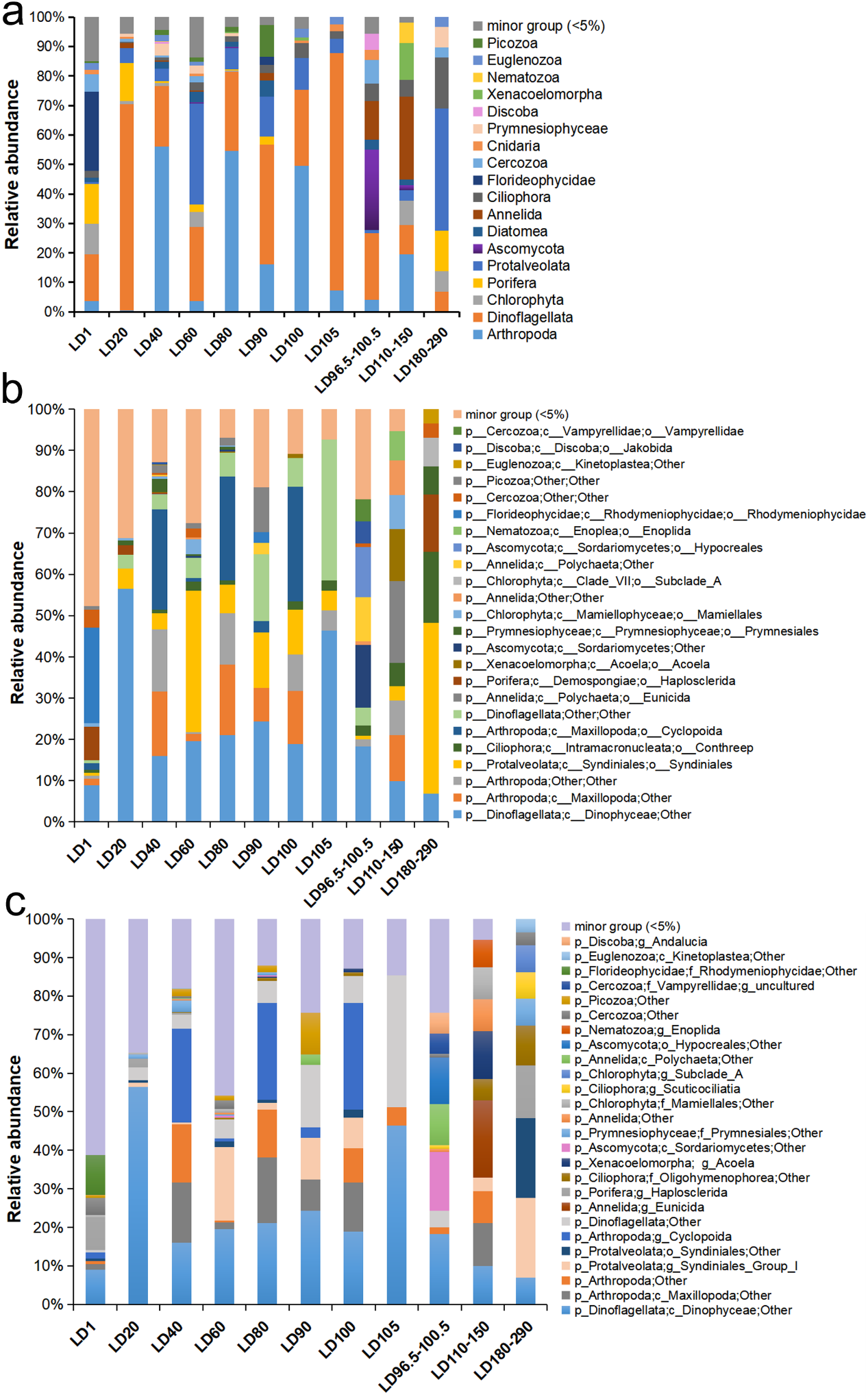


**Supplementary Figure 7** Eukaryotic community structures based on the 18S V4 miTags from 21 metagenomes at different levels. a, Eukaryotic community structures based on the 18S V4 miTags at phylum level. b, Eukaryotic community structures based on the 18S V4 miTags at order level. c, Eukaryotic community structures based on the 18S V4 miTags at genus level. The phyla, orders, and genera that accounted for less than 5% of the OTUs for metagenomes were grouped into “minor group”. Sampling depth (m) is indicated by the number or number range in the IDs that are described in Table S1.
